# A new sequential animal model for infection-related non-unions with segmental bone defect

**DOI:** 10.1186/s12891-020-03355-6

**Published:** 2020-05-27

**Authors:** Lars Helbig, Thorsten Guehring, Nadine Titze, Dennis Nurjadi, Robert Sonntag, Jonas Armbruster, Britt Wildemann, Gerhard Schmidmaier, Alfred Paul Gruetzner, Holger Freischmidt

**Affiliations:** 1 Clinic for Orthopedics and Trauma Surgery, Center for Orthopedics, Trauma Surgery and Spinal Cord Injury, Heidelberg University Hospital, Schlierbacher Landstrasse 200a, 69118 Heidelberg, Germany; 2 Arcus Sportklinik Pforzheim, Rastatterstr. 17-19, 75179 Pforzheim, Germany; 3 Unfallklinik Ludwigshafen, Klinik für Unfallchirurgie und Orthopädie, Ludwig-Guttmann-Strasse 13, 67071 Ludwigshafen, Germany; 4 Department of Infectious Diseases Medical Microbiology and Hygiene, Heidelberg University Hospital, Im Neuenheimer Feld 324, 69120 Heidelberg, Germany; 5 Laboratory of Biomechanics and Implant Research, Clinic for Orthopedics and Trauma Surgery, Heidelberg University Hospital, Schlierbacher Landstrasse 200a, 69118 Heidelberg, Germany; 6 Department of Trauma, Hand and Reconstructive Surgery, Experimental Trauma Surgery, Universitätsklinikum Jena, 07747 Jena, Germany; 7 Julius Wolff Institute, Charité—Universitätsmedizin Berlin, Corporate Member of Freie Universität Berlin, Humboldt-Universität zu Berlin, and Berlin Institute of Health, 13353 Berlin, Germany

**Keywords:** Animal model, Rat, Delayed osseous union, Non-union, Osteitis, Biomechanical testing, Micro-CT, Plate osteosynthesis, Two stage procedure

## Abstract

**Background:**

The treatment of fracture-related infections (FRI) is still a challenge for orthopedic surgeons. The prevalence of FRI is particularly high in open fractures with extensive soft-tissue damage. This study aimed to develop a new two-step animal model for non-unions with segmental bone defects, which could be used to evaluate new innovative bone substitutes to improve the therapeutic options in humans with FRI and bone defects.

**Methods:**

After randomization to infected or non-infected groups, 30 Sprague-Dawley rats underwent a transverse osteotomy of the mid-shaft femur with a 5 mm defect. Additionally, the periosteum at the fracture zone was cauterized at both sides. After intramedullary inoculation with 10^3^ CFU *Staphylococcus aureus* (infected group) or PBS (non-infected group), a fracture stabilization was done by intramedullary K-wires. After 5 weeks, the bone healing process was evaluated, and revision surgery was performed in order to obtain increased bone healing. The initial K-wires were removed, and debridement of the osteotomy-gap was done followed by a more stable re-osteosynthesis with an angle-stable plate. After further 8 weeks all rats were euthanized and the bone consolidation was tested biomechanically and the callus formation quantitatively by micro-CT analysis.

**Results:**

We developed and presented a new two-stage non-union animal model through a targeted *S. aureus* infection. After 5 weeks, all animals showed a non-union irrespective of assignment to the infected and non-infected group. Lane and Sandhu score showed a higher callus formation in the infected group. In all infected animals, the inoculated *S. aureus* strain was detected in the revision surgery. The second surgery did not improve bone healing, as shown by the Lane Sandhu score and in the μ-CT analysis. Similarly, biomechanical testing showed in both groups a significantly lower maximum torque as compared to the contralateral side (*p* < 0.0001).

**Conclusions:**

We were able to successfully develop a new two-stage non-union animal model, which reflects a genuine clinical situation of an infection-related non-union model with segmental bone defects. This model could be used to evaluate various therapeutic anti-infectious and osteoinductive strategies in FRIs.

## Background

In recent decades, our understanding of bone healing has grown rapidly. We have learned that bone healing is a complicated multifactorial process [1]. Experimental and clinical studies on osteomyelitis in recent years have improved our understanding of the pathophysiology of chronic bone infections. This is the basis for the development of new innovative therapeutic strategies for non-unions with segmental bone defects [2].

Nevertheless, the treatment of non-healing bone defects, especially those secondary to infections, is still a great challenge for orthopedic surgeons. Treatment often lasts months to years and involves radical and repeated surgical debridement in combination with intensive antibiotic treatment [3]. Recurrent infections are not uncommon and can lead to the loss of the affected extremity, which has enormous professional, social, financial, and familiar consequences for the patients [3].

The incidence of infections and osteomyelitis after complex fractures is high, ranging from 1 to 55% in primary fracture stabilization, especially in open fractures with extensive tissue damage [4–8]. *Staphylococcus aureus* is the most common pathogen associated with infected metal implants [9–11]. Delayed bone healing due to infection is often associated with large bone defects. The current gold standard, after eradication of the infection, is an autologous bone graft. However, this is only available to a limited extent, making this therapeutic strategy unsuitable for segmental bone defects [12].

Thus, there is a growing need for bone graft substitutes, including those being developed to be applied together with new strategies of bone regeneration such as tissue engineering and cell-based approaches as well as the need for bone graft substitutes with antibacterial effects [13]. However, a clear treatment algorithm in clinical practice may be difficult to follow due to the heterogeneous patient population. Therefore, a standardized animal model may deliver the necessary evidence to improve current treatment algorithms. So far, a multi-stage animal model that can be realistically transferred to the clinical situation in humans has not been established. Such a model would be of great significance in the development of novel or optimization of therapeutic concepts for infected and non-infected non-unions.

The goal of this study was to develop and establish a new two-step sequential animal model of delayed fracture healing using *S. aureus* as an infecting agent in order to investigate innovative treatment concepts subsequently.

## Methods

### Preparation of infecting agent

*S. aureus* subsp. aureus ATCC® 49230™ was used for the in vivo infection. On the day of the infection, *S. aureus* was cultured in tryptic soy broth (TSB) at 37 °C with 5% CO_2_ under constant shaking (200 rpm) and harvested at the mid-log phase. After washing with sterile phosphate-buffered saline (PBS), the bacterial pellet was resuspended in sterile PBS and adjusted to 0.5 McFarland (equivalent to 1.5 × 10^8^ CFU/ml) and diluted to 1 × 10^5^ CFU/ml. 10 μl of the diluted bacterial suspension containing 10^3^ CFU was used for in vivo infection, as described below.

### Implant

Stainless steel Kirschner wires, 1.2–1.6 mm in diameter (Synthes GmbH, Umkirch, Germany, diameter of the wire adjusted to the diameter of the femur), were used for the first osteosynthesis and an angle-stable plate-osteosynthesis (RISystem AG, Davos, Switzerland) for the second step procedure.

The following groups were examined (*n* = 15 animals each group):

**Table:** 

Non-infected group		Infected group
		*S. aureus* with 10^3^ CFU
K-wire osteosynthesis		K-wire osteosynthesis
	after five weeks	
Debridement		Debridement
+		+
Re-osteosynthesis		Re-osteosynthesis
with an angle stable plate		with an angle stable plate

### Animals, operative procedure, and osteotomy model

All experiments were approved by the Animal Experimentation Ethics Committee of the Regierungspräsidium Karlsruhe; Abteilung 3; Baden-Württemberg (35–9185.81/G-155/17). Thirty female, 3-months-old Sprague–Dawley rats (Charles River, Germany) with an average weight of 277 g were divided into two study groups with 15 rats per group. They were housed in standard laboratory conditions (12-h light; 12-h dark cycle, room air temperature 22–24 °C, three rats per cage) and had free access to tap water and food pellets. Surgery was performed under general anesthesia by weight-adopted intraperitoneal injection of medetomidine (Dorbene Vet 1 mg/ml), midazolam (Midazolam HEXAL 5 mg/1 ml) and fentanyl (Fentadon Dechra 50 μg/ml).

**Fig. 1:**
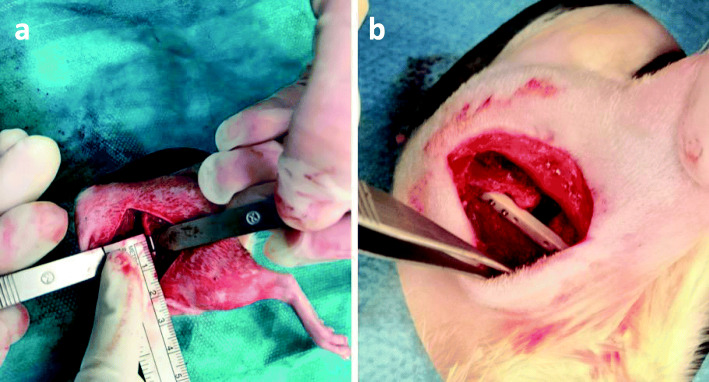
Representative intraoperative situation during the first surgery after k-wire osteosynthesis (**a**) of the femoral critical size defect (CSD) and the intraoperative situation during the second surgery 5 weeks later after the angle-stable plate osteosynthesis (**b**) of the femoral critical size defect

During the first surgery, the left hind leg was shaved and disinfected with alcohol. The animals were placed on sterile drapes and the bodies were covered with sterile sheets. The lateral approach to the mid-shaft femur was performed for the two-step surgery. Skin and fascia at the anterolateral aspect of the femur were incised over three cm in length. The entire length of the femoral shaft was exposed by separating the vastus lateralis and biceps femoris muscles. All attached muscles and periosteum were stripped from the shaft. Afterwards a 5-mm mid-diaphyseal full- thickness osteotomy was performed using a diamond disk (Dremel, Racine, USA). The medullary cavity of the femur was reamed with a 1.2–1.6 mm stainless steel Kirschner-wire (Synthes GmbH, Umkirch, Germany) up to the distal and proximal part of the medullar cavity. After removal, 10 μl of bacterial suspension containing 10^3^ CFU of *S. aureus* (ATCC® 49,230™) or 10 μl PBS were injected into the medullary cavity according to study groups. After contamination, the osteotomy was stabilized intramedullary with a 1.2–1.6 mm stainless steel Kirschner-wire (Synthes GmbH, Umkirch, Germany) (Fig. 1a) according to the diameter of the medullary canal. Thereby, an intentionally unstable rotational situation and thus, unstable osteosynthesis was created. Soft tissue was irrigated, and skin, fascia and muscles were sutured in an intracutaneous technique (Resolon® 4/0 Ethicon, Norderstedt, Germany). In addition, the wound margins were adapted with skin clamps (Autoclip Clips, Heidelberg, Germany).

All animals received second surgery 5 weeks after the first surgery. The skin incision was placed over the scar of the previous suturing. The k-wire was carefully removed after radical debridement of the non-union and the medullar cavity was irrigated with sterile saline. Afterwards, the osteotomy region was thoroughly debrided and small tissue samples were removed for microbiological investigations. After debridement, an angle-stable polyacetyl plate (length, 25 mm; width, 4 mm; height, 4 mm; RISystem AG, Davos, Switzerland) was placed on the anterolateral surface of the femur. The angle-stable plate had eight predrilled holes to accommodate eight angle-stable cortex screws. Then six pilot holes (three distal and three proximal to the osteotomy) were drilled through the predrilled holes of the plate and both cortices of the femur. After each pilot hole was drilled, an angle-stable screw was inserted through the plate and both cortices (Fig. 1b). After placement of the six angle-stable screws, soft tissue was irrigated again, and skin, fascia and muscles were sutured in an intracutaneous technique (Resolon® 4/0 Ethicon, Norderstedt, Germany). In addition, the wound margins were adapted with skin clamps (Autoclip Clips, Heidelberg, Germany).

### Follow-up

On the day of surgery and regularly throughout the observation period, body weight and body temperature were measured. The clinical condition of the animals was evaluated. All animals received buprenorphine (0.3 mg/ml bw; Buprenovet®) as analgesic medication perioperatively. Animals were followed up for 8 weeks after secondary surgery and were then sacrificed.

### μCT scan evaluation

μCT scans of the left femora were taken immediately before and 8 weeks after the second surgery (angle-stable plate osteosynthesis) at the endpoint. All animals were scanned with the SkyScan 1076 in-vivo micro-computed tomography scanner (Brucker micro-CT, Belgium) as previously described [14]. The femora were scanned with a scan orbit of 360 degrees, an isotropic pixel size of 18 μm and energy settings of 100 kV (voltage), 280 ms (exposure time) and 100 μA (current) through a 1.0-mm aluminum filter. The rotation step was 0.4° and the frame averaging was 6. Image reconstruction, including ring artefact reduction (9/20), beam hardening correction (30%) and smoothing (1/10) was performed using the SkyScan NRecon software (v.1.6.9.8, Brucker microCT, Belgium).

Qualitative evaluation of the datasets was conducted by simultaneously viewing multiple orthogonal slices in SkyScan DataViewer (v.1.5.2.4, Brucker microCT, Belgium). Additionally, new bone formation at the osteotomy site, bridging of the osteotomy and bone remodeling were analyzed by two independent observers adopted from the scoring system of Lane and Sandhu and given points from 0 to 4 for each of the three aspects (Table 1) [15]. Hence, the highest possible score was 12 points. The scoring system was used as “bone-healing-score” as previously described [14]. The modified score of An and Friedman was used to determine the grade of osteitis at the endpoint [16, 17], analyzed by two independent observers according to the scoring system as displayed in Table 2. The score of An and Friedman is a radiographic scoring system for assessing the development and progression of osteomyelitis in a rabbit model. A modified form of this score was used in the study as “bone-infection-score” as previously described [14]. Six characteristic parameters were analyzed (Table 2). For evaluation of the parameter 1 to 4, the femur was divided into three regions of interest (ROI) – proximal end, critical size defect (CSD) and distal end, and a score from 0 (absent), 1 (mild), 2 (moderate) to 3 (severe) was given to each ROI. For parameters 5 and 6 the whole femur was graded with a score of either 0 (absent) or 1 (present). Therefore, the highest possible score was 38.

**Table 1:** Modified score of Lane and Sandhu (radiographic criteria)

	***Score***
**Bone formation**	
No evidence of bone formation	**0**
Bone formation (25% of the gap)	**1**
Bone formation (50% of the gap)	**2**
Bone formation (75% of the gap)	**3**
Bone formation (100% of the gap)	**4**
**Bridging**	
No evidence of bridging	**0**
Bridging (25% of the gap)	**1**
Bridging (50% of the gap)	**2**
Bridging (75% of the gap)	**3**
Bridging (100% of the gap)	**4**
**Bone remodeling**	
No evidence of bone remodeling	**0**
Bone remodeling (25% of the gap)	**1**
Bone remodeling (50% of the gap)	**2**
Bone remodeling (75% of the gap)	**3**
Bone remodeling (100% of the gap)	**4**

**Table 2:** Modified score of An and Friedman (radiographic criteria)

***Variable***	***Definition***	***Score***
**1. Periosteal reaction**	absent	0
	mild	1
	moderate	2
	severe	3
**2. Osteolysis**	absent	0
	mild	1
	moderate	2
	severe	3
**3. Deformity**	absent	0
	mild	1
	moderate	2
	severe	3
**4. General impression**	absent	0
	mild	1
	moderate	2
	severe	3
**5. Sequester formation**	absent	0
	present	1
**6. Spontaneous fracture**	absent	0
	present	1

Further quantitative evaluation was performed using the SkyScan CTAnalyzer (v.1.13.21, Brucker microCT, Belgium). The region of interest included a bone area of 6 mm over the osteotomy area. The middle of the defect gap was determined for each bone individually and starting from there 3 mm in proximal and distal direction. That area contains 175 images in each direction. The extend of the VOI (volume of interest) in the transversal plane has been defined semi-manually by the region of interest (ROI) function. At intervals of about 20 slices a ROI was drawn manually around the bone (function: polygonal ROI) including a narrow seam of soft tissue. For the slices in between, the program automatically calculates the extent of the ROI (function: interpolated ROI). The following parameters were used for bone morphometry of the VOI for each animal at the endpoint after 13 weeks: bone volume, bone surface, bone density, bone surface density, trabecular number and total porosity.

### Body weight and body temperature

Rectal body temperature was measured immediately before and after the first and second surgery and after one, five, six and 13 weeks. Body weight was determined with a precision scale until they reached the initial weight. The animals were explored for clinical signs of infection (swelling, reddening, impairment of wound healing, loss of passive motion in the left hind leg).

### Microbiological evaluation

Microbiological samples were taken during the second surgery and after the animals were sacrificed. 10 μl of swabs and clinical samples were cultured on BD Columbia Agar with 5% Sheep Blood (Becton Dickinson, Heidelberg, Germany) at 37 °C with 5% CO_2_ for 24 h. Colonies with morphological consistency with *S. aureus* were confirmed by a slide agglutination test (Pastorex Staph Plus, Bio-rad, Germany), as described elsewhere [18]. Strain typing was performed by Sanger sequencing and comparison of the polymorphic protein A gene (spa typing) as previously described [18].

### Sacrifice

The animals were sacrificed under general anesthesia with CO_2_ in a sedation box. The left and the right femora of the hind legs were dissected under sterile conditions. The entire soft tissue was removed from bones. Small microbiological samples have been taken for investigations.

Afterwards, the bones were processed according to randomization for histological or biomechanical evaluation.

### Mechanical testing

The biomechanical torsional testing device was the same as previously described [14]. The experimental procedure was modified and adjusted. Both femora were dissected free from soft tissue and the plate osteosynthesis for biomechanical torsional testing (non-infected group: seven samples; infected group: seven). After dissection of the bones, the proximal and distal ends were placed into two embedding molds (Technovit 4071, Heraeus Kulzer GmbH, Germany) while using a fixture device to avoid any preloading of the bones prior to testing. The lower embedding mold was connected to a pivoted axis while rotation of the upper mold was restrained. A linear, constant rotation (20°/min) was applied by the biomechanical testing device while the resultant torque was recorded (8661–4500-V0200, Burster, Germany) until fracture occurred. For biomechanical testing a comparison with the contralateral femora of each animal was performed.

### Histology

Soft tissue and muscles were removed from the femora before they were prepared for histology by standard procedures (non-infected group: five samples; infected group: seven): after being fixed for 4 days in 4.5% paraformaldehyde (roti-Histofix, Roth, Karlsruhe, Germany) and decalcified for 3 weeks in ethylenediaminetetraacetic (Entkalker soft, Roth, Karlsruhe, Germany) the plates and screws were withdrawn. The samples went through a graded alcohol series for dehydration and finally embedded in paraffin. The femora were cut until the center of the sample and the sections were stained with alcianblue, iron hematoxylin, brillant-crocein-fuchsin-acid and safran (Pentachrom: Chroma-Waldeck GmbH & co. KG, Münster, Germany)statistics

The primary outcome was measured according to the maximal torque in Nm in biomechanical analysis. Secondary outcome measures were the score according to An and Friedman and the data of the μ-CT analysis. Complete data sets were available for 26 animals. Mean and standard deviation (SD) were calculated. With the D’Agostino-Pearson test all data were checked for normal distribution. Differences between continuous variables (e.g. infected vs. non-infected group) was tested with an unpaired t-test. Comparison of more than two groups was performed using one-way analysis of variance (ANOVA) for independent samples and balanced according to Bonferroni. The post hoc power analysis for the ANOVA revealed an effect size f of 0.904 and an actual power of 0.971 for the biomechanical evaluations as the primary end point. All tests were two-sided and a *p* value ≤0.05 was considered significant. Statistical analysis was performed using GraphPad Prism version 8.00 for Mac, GraphPad Software, San Diego California USA for the bar graphs.

## Results

### Failure parameters

One animal of each group died during the anesthesia immediately after the second surgery for unclear reasons. Two animals from the non-infected group had to be excluded due to a secondary bone infection, as determined by a positive microbiological result and an An and Friedman score ≥ 19. The final animal population in the non-infected group was 12 and 14 in the infected group, respectively.

### Body weight

The bodyweight within 5 days after the first surgery increased significantly lower in the infected group as compared to the non-infected group (*p* < 0.021, unpaired t-test). No significant differences in body weight was detected between both groups after the second surgery and at the time of sacrifice (*p* > 0.05, unpaired t-test) (data not shown).

### Microbiological evaluation / cultures of implants / wound infection

After sacrifice, *S. aureus* could be cultured in all microbiological samples of the infected group (*n* = 14). A septic loosening (positive bacteriology *and* An and Friedman score ≥ 19) of the osteosynthesis was observed in 79% of the infected group. Interestingly, *S. aureus* could be cultured in six animals (43%) of the non-infected group (*n* = 14) at the time of sacrifice. Of those animals only two (14%) showed clear clinical or radiological signs of bone infection (An and Friedman score ≥ 19) and were consequently excluded from the evaluation. Molecular characterization by *spa* typing confirmed that all infections in the non-infected group was due to a different *S. aureus* strain with the *spa* type t1754 and not from the infecting agent (*spa* type t021) used in the infected group.

In 14% of the non-infected group, an aseptic loosening was observed, as defined by negative bacteriology, missing radiological and histological signs of infection (An and Friedman score ≤ 19).

In 21% of the non-infected group and 79% of the infected group, a new wound examination with debridement and new skin suture was indicated during the follow up due to wound infection.

### μCT scan examinations and modified x-ray scores according to “Lane and Sandhu” and “An and Friedman”

**Fig. 2:**
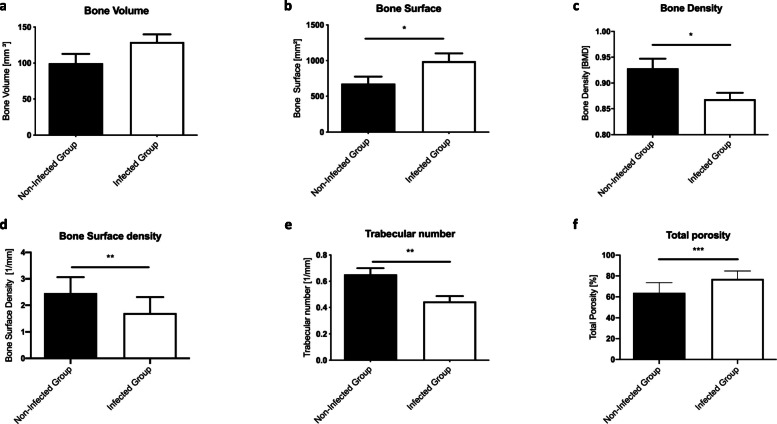
Quantitative micro-CT evaluation of bone volume (mm^3^) (**a**), bone surface (mm^2^) (**b**), bone density (BMD)(**c**), bone surface density (1/mm) (**d**), trabecular number (1/mm) (**e**) and total porosity (%) (**f**) of the two groups at the endpoint. Lines with asterisk depict significant differences between groups (* *p* < 0.05)

Eight weeks after the second surgery, the infected group (*n* = 14) showed lower bone volume compared with the non-infected group (*n* = 12) (*p* = 0.0878). However, the bone surface was significantly increased in the infected group (*p* = 0.0429). At the time point of sacrifice, eight weeks after the second surgery, the infected group showed significant reduced bone density (*p* = 0.012, unpaired t-test), bone surface density (*p* = 0.0043) and trabecular number (*p* = 0.0028), whereas the trabecular separation (*p* = 0.0008) and total porosity (*p* = 0.0005) were significantly increased, compared to the non-infected group (Fig. 2a-f).

**Fig. 3:**
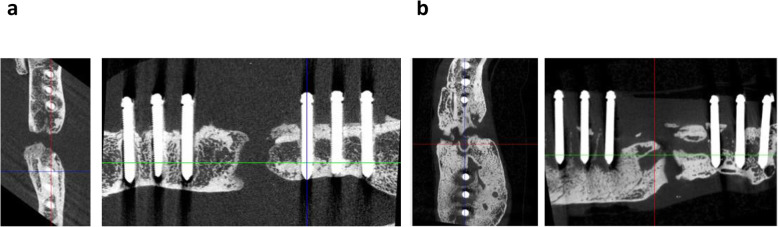
Micro computed tomography (micro-CT) of the left femora of Sprague-Dawley rats at the endpoint. Osteotomy with segmental defect, bacterial infection or sham infection, were performed 13 weeks before; angle-stable osteosynthesis with an angle-stable plate was done 8 weeks before analysis. No consolidation of the segmental defect is recognizable in the non-infected group (**a**) and the infected group (**b**). Hypertrophic callus formation, reduction of bone density, change of the trabecular structures and peri-implant loosening were detected in the infected group (**b**)

The critical size defect was not bridged after 5 weeks in all animals of both groups (Fig. 3a; b).

**Fig. 4:**
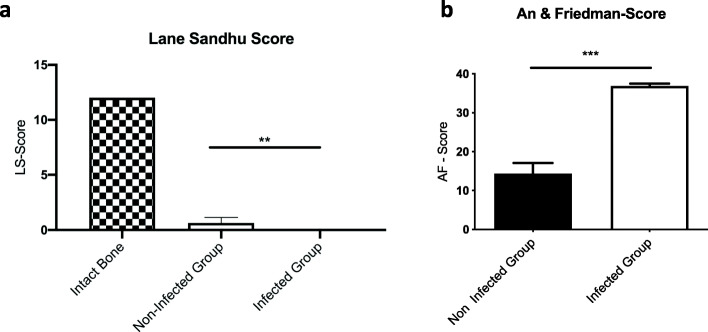
Outcome of (**a**) the Lane & Sandhu score with a maximal score of 12 points and (**b**) the modified An & Friedman score with six characteristic parameters for a maximal score of 38 points. Both scores are significantly different between the non-infected and the infected group. Lines with asterisk depict significant differences between groups (* *p* < 0.05)

According to Lane and Sandhu’s modified x-ray score, the median score in the non-infected group was higher than in the infected group. In both groups, no healing could be observed (Fig. 4a).

The modified An and Friedman score was significantly higher in the infected group as compared to the non-infected group at the time point of revision surgery (*p* < 0.0001) and at euthanasia (*p* < 0.0001) (Fig. 4B).

### Mechanical testing at sacrifice (eight weeks after plate osteosynthesis)

**Fig. 5:**
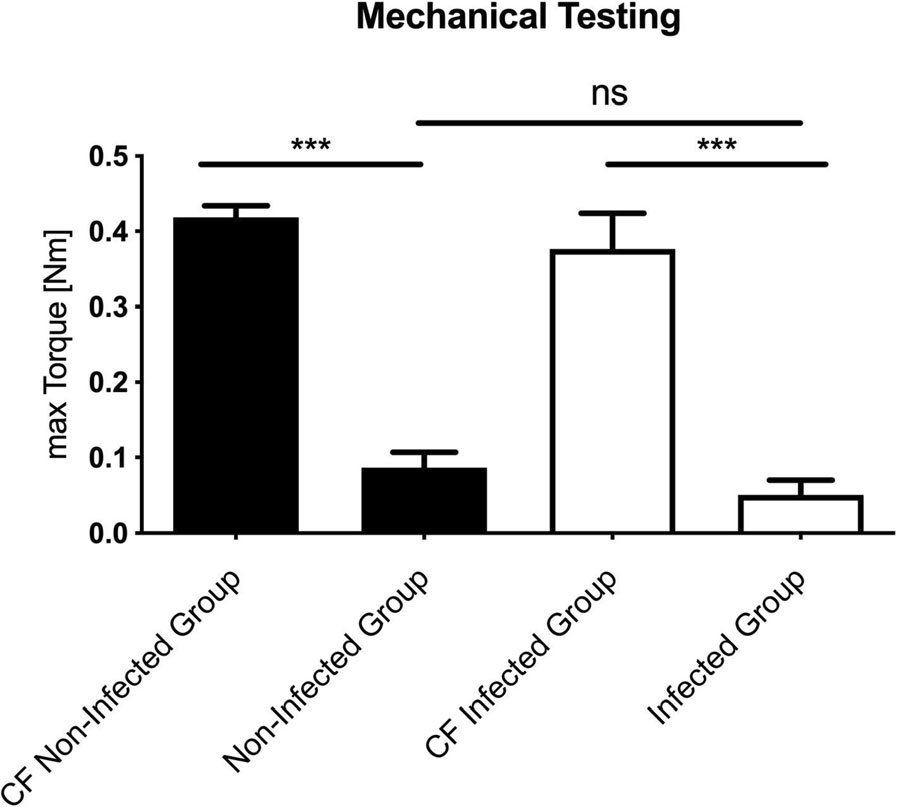
Mechanical testing results of the femora. The maximum re-fracture torque (Nm) is significantly different between the left and contralateral femur (CF). No significant differences (ns) of maximum re-fracture torque (Nm) were detected between the non-infected and infected group at the endpoint. Lines with asterisk depict significant differences between groups (* *p* < 0.001)

After sacrificing, the plate osteosynthesis was carefully removed and the specimen underwent biomechanical investigation. No difference between the maximum torque (Nm) of the non-unions in the non-infected group could be detected as compared with the non-unions in the infected group (*p* = 0.381; ANOVA). The tested left femur of the non-infected group demonstrated an average maximum torque of 0.087 +/− 0.054 Nm. The average maximum torque of the infected group was 0.05 +/− 0.051 Nm. Maximum torque (Nm) of the intact contralateral side was significantly higher in both groups compared to the right defect femur (*p* < 0.0001, ANOVA). The differences between the non-infected and infected group on the contralateral side revealed no statistical significance (*p* = 0.309 ANOVA) (Fig. 5).

### Histology

**Fig. 6:**
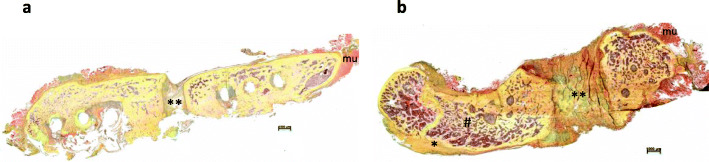
Overview and magnification (2.5x) of the fracture region stained with Pentachrome (mineralized bone: dark yellow; mineralized cartilage: blue-green; fibrous tissue: light yellow; muscles: red): In the infected group (**b**) the callus is hypertrophic mineralized (*), the bone density is reduced (#). No fracture healing is visible in the non-infected (**a**) and infected (**b**) group with fibrous tissue and cartilage (**) filling the gap. **a** non-infected group, **b** infected group; mineralized bone: *; fibrous tissue and cartilage: **; muscles: mu

Figure 6 illustrates that independent of infection or not the animals developed a clear non-union. In the infected non-unions, a hypertrophic callus formation and a reduction of bone density as well as a change of the trabecular structures were visible, as similarly described in the μ-CT evaluation.

## Discussion

The treatment of critical size bone defects resulting from trauma or posttraumatic osteomyelitis is still very challenging for the orthopedic surgeon and has major socio-economic consequences [3, 19, 20]. *S. aureus* is the most common bacterium in bone infection after open fractures [10, 21, 22]. Moreover, *S. aureus* infection is an established and well-investigated animal model to study osteomyelitis [4, 23, 24]. Bone healing on critical size defects, which is defined as bone loss greater than 50% of the circumference of the bone, is investigated in many animal models too [25–27]. However, there is little evidence and a lack of consensus around the definition of critical-sized bone defects [27]. Several other animal studies have also investigated bone healing combined with osteitis [28–31]. Most of these models investigate acute infections [29–31]. In contrast to our two-stage animal model, these models performed inoculation with the agents and the treatment of the impaired bone healing at the same time [29–31]. The two-stage procedure with inoculation and treatment at different time points demonstrates increased clinical relevance. Other models initiated chronic osteomyelitis after intravenous inoculation of *S. aureus* [32]. It’s obvious that the systemic intravenous inoculation cannot guarantee a reliable and reproducible local bone infection in the site of interest [33]. This is one of the major advantages of our model. It guarantees a reliable and reproducible bone infection without further systemic infections and a consecutive immunosuppression. Other models used ectopic bone xenografts, which were inoculated with *S. aureus* [34]. But the use of bone xenografts shows only limited clinical relevance in accordance to the human situation [32, 34]. Great bone loss is often associated with chronic osteitis. Therefore, we aimed to develop a new sequential animal model that combined both aspects of bone defects with a chronic local bone infection.

To develop a standardized animal defect model, which allows the study of new innovative bone graft substitutes for non-healing bone defects, we preferred an osteotomy rather than a more difficult to reproduce fracture model as in the present experiment. In our previous experiments, we used a fracture model with an established fracture device to create a typical closed fracture [14, 23, 35]. But the major limitation was that the individual fracture pattern performed with the fracture device was complex and difficult to predict [23]. For this reason, in the literature exists several animal bone defect models, which evaluate bone healing using osteotomy [28, 30, 31]. The research group of Windolf et al. described a mice model using a locking plate fracture stabilization after performing an osteotomy [30, 31]. Schindeler and co-workers demonstrated an infected open fracture model with *S. aureus* in rats [29] and Chen et al. [28] performed a segmental femur defect model by creating a chronic infection with stabilization by plate and k-wires after creating an osteotomy. Brunotte et al. developed a two-stage-revision model in implant-related methicillin-resistant *S. aureus* bone infection [36]. In contrast to our model, a two-stage intramedullary K-wire-osteosynthesis was performed in his animal model. To the best of our knowledge, our study is the first two-step animal model for infected and non-infected non-unions with segmental bone defect, which could be used to test different new innovative bone substitutes and which best reflect the human situation of fracture related infections using a combination of an intramedullary nail osteosynthesis and an angle-stable plate osteosynthesis.

Kaspar and colleagues demonstrated in their osteotomy-model a biomechanically stable fracture bridging using a rigid custom-made external fixator osteosynthesis in rats after 56 days [37]. In contrast to our study, a segmental bone defect was not used. In our model, the second surgery using an angle-stable plate osteosynthesis was performed to obtain more stability at the defect zone, as typically done during revision surgery in the human situation. However, in contrast to the results of Kaspar et al. [37], in our study the bone defect persisted without bridging 8 weeks after revision surgery with an angle-stable plate osteosynthesis in our μCT scan and biomechanical evaluations in both groups, which is concordant to segmental bone defects. Significant differences between the infected and non-infected groups was not observed. In μCT data, bone volume tends to increase in the infected group, whereas bone surface is significantly higher compared to the non-infected group. This observation can be explained by the significantly increased porosity as well as by the significantly reduced density and trabecular structure in infected animals. These findings are also consistent with the results from the Lane and Sandhu score and the biomechanical testing. The An and Friedman score is a well-proven radiographic scoring system for assessing the development and progression of osteomyelitis in a rabbit model [14, 16, 23]. Highly significant differences between the two groups could be detected at the endpoint. The infection-related bone changes are consistent among small or large animal models, and are similar to those seen in patients [38–40].

Additionally, at the time of sacrifice, the microbiological examination identified the inoculated *S. aureus* strain in all infected animals, resulting in a septic loosening of the plate osteosynthesis in 79% of the infected cases. In these cases, the infected non-unions could be verified μ-computertomographically, biomechanically and clinically. In the non-infected group, *S. aureus* was found in 43% of the samples. Only two animals in this group showed clear clinical and radiological signs of infection, which may be due to secondary post intervention infections. The risk of secondary infections after internal fixation in humans is between 0.4% and up to 16.1% according to the type of fracture [41, 42]. A possible explanation for the high rate of positivity in the non-infected group could be contamination in several processes, such as initial infection, surgery and (postmortem) sampling. Also, natural colonization of the nares and mucosal surfaces of the rats by *S. aureus* cannot be ruled out [43], as all *S. aureus* isolates from non-infected animals belong to the same clonal group (*spa* type t1754), but a different clonal group from the infecting agent (*spa* type t021) used initially. In addition, the t1754 *S. aureus* lineage has been attributed to zoonotic *S. aureus* colonization [44]. Moreover, manipulation of surgical wounds by the animals was observed and hence secondary wound contamination with increasing bone infections is another possible pathophysiological route. In another 14% of the non-infected animals an aseptic loosening of the osteosynthesis occurred. This could be attributed to insufficient stability despite the secondary stabilization with a plate. Another aspect includes the fact that immediately after the second surgery, the animals are full weight-bearing, as a partial weight-bearing is not possible in rats. In conclusion, the pathophysiological route of infection with insufficient bone healing in the non-infected group may be heterogenous and could include mechanical as well as biological aspects. The shown picture of the pathophysiology of non-healing in this group may perfectly resemble the clinical situation of a fracture-related infection [45–47].

In summary, this sequential animal model is reproducible and could be used to test different bone substitutes and osteoinductive agents to improve the therapeutic options in humans with fracture-related infections and bone defects.

## Conclusion

Non-unions are often associated with chronic infections and are difficult to treat. We were able to develop a two-stage animal model of clinical relevance, reflecting the in vivo human situation. This allows different therapeutic approaches for fracture-related infections and bone defects to be tested in an in vivo experimental chronic infection model in rats. Thus, allowing further investigations of osteoinductive and anti-infective potential of different bone substitutes as well as their ability to remodel impaired bone.

## Data Availability

The datasets used and analyzed during the current study are available from the corresponding author on reasonable request.

## Abbreviations

CFU: Colony-Forming Unit
CT: Computer Tomography
FRI: Fracture Related Infections
K-wire: Kirschner-wire
PBS: Phosphate-Buffered Saline
TSB: Tryptic Soy Broth
*S. aureus*: *Staphylococcus aureus*
ROI: Region of interest
VOI: Volume of interest
